# Abrogation of the Twin Arginine Transport System in *Salmonella enterica* Serovar Typhimurium Leads to Colonization Defects during Infection

**DOI:** 10.1371/journal.pone.0015800

**Published:** 2011-01-26

**Authors:** M. Megan Reynolds, Lydia Bogomolnaya, Jinbai Guo, Lindsay Aldrich, Danial Bokhari, Carlos A. Santiviago, Michael McClelland, Helene Andrews-Polymenis

**Affiliations:** 1 Department of Microbial and Molecular Pathogenesis, College of Medicine, Texas A & M University System Health Science Center, College Station, Texas, United States of America; 2 Departamento de Bioquímica y Biología Molecular, Facultad de Ciencias Químicas y Farmacéuticas, Universidad de Chile, Santiago, Chile; 3 Vaccine Research Institute of San Diego, San Diego, California, United States of America; 4 Department of Pathology and Laboratory Medicine, College of Health Sciences, University of California Irvine, Irvine, California, United States of America; University of Osnabrueck, Germany

## Abstract

TatC (STM3975) is a highly conserved component of the Twin Arginine Transport (Tat) systems that is required for transport of folded proteins across the inner membrane in gram-negative bacteria. We previously identified a Δ*tatC* mutant as defective in competitive infections with wild type ATCC14028 during systemic infection of *Salmonella-*susceptible BALB/c mice. Here we confirm these results and show that the Δ*tatC* mutant is internalized poorly by cultured J774-A.1 mouse macrophages a phenotype that may be related to the systemic infection defect. This mutant is also defective for short-term intestinal and systemic colonization after oral infection of BALB/c mice and is shed in reduced numbers in feces from orally infected *Salmonella*-resistant (CBA/J) mice. We show that the *ΔtatC* mutant is highly sensitive to bile acids perhaps resulting in the defect in intestinal infection that we observe. Finally, the Δ*tatC* mutant has an unusual combination of motility phenotypes in *Salmonella*; it is severely defective for swimming motility but is able to swarm well. The Δ*tatC* mutant has a lower amount of flagellin on the bacterial surface during swimming motility but normal levels under swarming conditions.

## Introduction

The Twin Arginine Transport (TAT) system is required in many organisms for the transport of folded proteins from the bacterial cytoplasm into the periplasm. Mutations in Tat systems in pathogenic bacteria, including *E. coli* O157:H7, *Yersinia pseudotuburculosis* and *Pseudomonas aeruginosa*, are known to have effects on many processes including virulence, toxin secretion, swimming and/or swarming motility, chemotaxis, and biofilm formation [1–0].

The *Salmonella enterica* serotype Typhimurium twin arginine transport system encoded by the TAT operon has ∼80% identity to the TAT operon in *E. coli*. This ubiquitous system transports substrates containing a positively charged N-terminus with the canonical signal sequence S/T-R-R-x-F-L-K prior to a hydrophobic core and a peptidase cleavage site [3], [11], [12]. The TAT secretion system machinery includes 3 major components, TatA, B and C. TatA is found in molar excess and is responsible for formation of the pore that allows folded proteins traverse the membrane [13]. TatB plays a role in recognition of the signal sequence of substrates and interaction of TatA with TatC [14]. TatC is the largest component of this transport apparatus [15], [16] and is highly conserved, especially at the termini and other cytoplasmic domains [17]–[9]. Initial docking of the signal sequence occurs via TatC [20]. While many of the substrates of the TAT system are cofactor-containing redox enzymes, the particular substrates transported by the TAT system in different organisms are quite variable. The TAT system of *Salmonella* is predicted to transport approximately 30 proteins into the periplasm, including one substrate that lacks a twin-arginine and is *Salmonella* specific, TtrB [21], [22].

We became interested in the TAT system in *Salmonella*, when we identified a Δ*tatC* mutant as negatively selected in a previous screen for mutants under selection during systemic infection [23]. In the current study, we explore the effects of deletion of *tatC* in *Salmonella enterica* serovar Typhimurium ATCC14028 during infection. We confirm that our Δ*tatC* mutant is impaired for colonization of systemic sites after intraperitoneal delivery in *Salmonella*-susceptible BALB/c mice, and is internalized by J774-A.1 murine macrophages in lower numbers than the isogenic wild type parental isolate. We also studied the ability of our Δ*tatC* mutant to colonize the intestinal tract and spread systemically after oral delivery and show that this mutant is highly sensitive to bile acids. Finally, we show that a Δ*tatC* mutant has an unusual motility defect; it is unable to swim yet swarms normally.

## Results

### Typhimurium Δ*tatC* mutant attenuated during murine systemic infection

**Figure 1:**
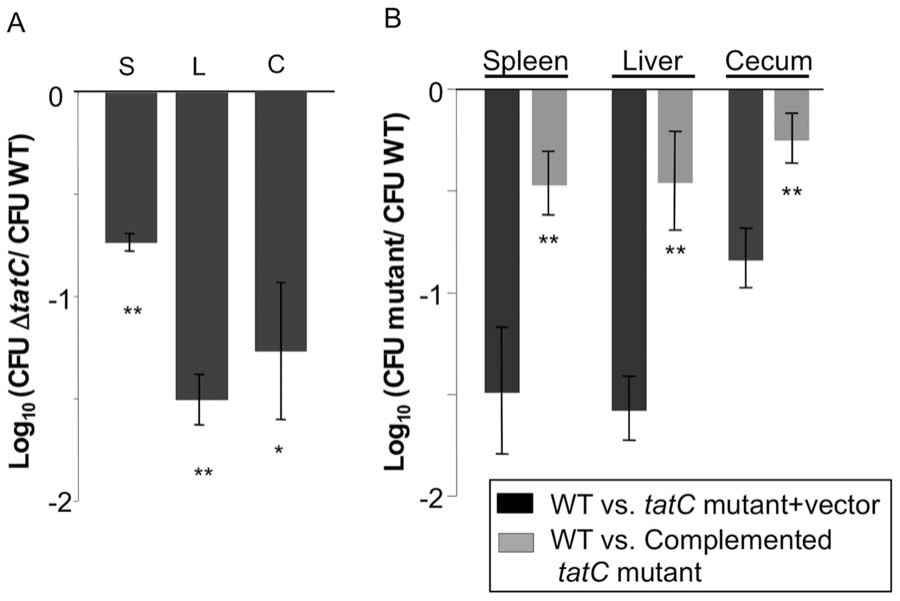
Δ*tatC* mutant colonizes *Salmonella*-susceptible mice poorly after intraperitoneal inoculation. (A) *Salmonella*-susceptible mice were infected with an equal ratio of HA431 (ATCC14028 Nal^R^ Δ*phoN:kan*) and HA473 (ATCC14028 Nal^R^ Δ*tatC::*Kan^R^) by intraperitoneal inoculation with ∼1×10^6^ CFU. Mice were humanely euthanized at 48 hours post infection and liver, spleen and cecum were collected, homogenized, serially diluted and plated on appropriate media to enumerate both infecting strains. Bars represent the geometric mean of the ratios of the infecting strains post infection, normalized to the ratio of the same in the inoculum, and converted logarithmically. Error bars denote standard error. Statistical significance was determined by Students t- test. *p<0.05, **p<0.001 (B) Competitive infections were performed with HA431 and either HA636 (Nal^R^ Δ*tatC::*Kan^R^ pBAD TOPO Carb^R^, empty vector control) (B, dark bars) or HA640 (Nal^R^ Δ*tatC::*Kan^R^ pBAD TOPO *tatC* Carb^R^, complementing plasmid) (B, light bars). Infections were performed as described above, and the CFU of the infecting strains was enumerated from infected organs as shown. Statistical significance was determined by Students t- test. **p<0.01.

We previously identified a large group of genes apparently required for maximal fitness of *Salmonella* during systemic infection in *Salmonella*-susceptible BALB/c mice and that had not already been described to have this phenotype [23]. This list included a deletion mutant in *tatC*. Here, we transduced this mutant to a clean genetic background to generate HA473, and we tested this strain in competitive infections with wild type ATCC14028 derivative HA431. We confirmed that the Δ*tatC* mutant has a reduced ability to colonize the liver and spleen after intraperitoneal infection (Figure 1A).

In order to link the defect in systemic infection to the mutation in *tatC*, we complemented our Δ*tatC* mutant *in trans* and tested colonization the complemented strain during competitive systemic infections. The Δ*tatC mutant* (HA636) bearing the empty vector, and the mutant complemented *in trans* with an intact copy of *tatC* (HA640) were used in competitive infections with our wild type (HA431). The mutant containing the empty vector maintained a colonization defect during infection (Figure 1B, dark grey bars). In contrast, the complemented mutant regained the ability to colonize systemic sites well (Figure 1B, light grey bars).

Infection by the intraperitoneal route allows the infecting strains bypass the intestinal tract, a niche that non-typhoidal *Salmonella* must successfully traverse during most naturally acquired infections. Thus, the Δ*tatC* mutant is defective for colonization and replication in systemic sites even when it is delivered by a route that bypasses the intestine and would typically lead to systemic infection.

### Δ*tatC* mutant in Typhimurium ATCC14028 is defective during infection of J774-A.1 macrophages

**Figure 2:**
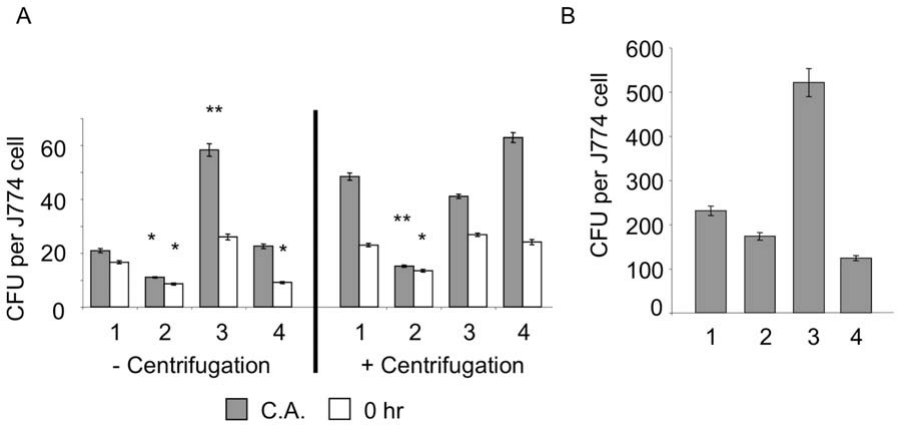
Δ*tatC* mutant is defective for invasion of macrophages. J774-A.1 macrophages were infected with either: (1) Wild type (HA420)(ATCC14028s Nal^R^), (2) Δ*tatC* mutant (HA473), (3) Δ*tatC* mutant complemented *in trans* with intact *tatC* (HA640), or (4) a deletion mutant in *invA* (HA458) and a standard gentamicin protection assay was performed either with or without centrifugation (to minimize the confounding effect of the motility defect of Δ*tatC).* Panel (A) Cell association and bacterial uptake by macrophages are shown. (B) Intracellular replication after 24 hours is shown. For each sample the geometric mean of triplicate samples from three independent experiments is shown. Error bars denote standard error, and statistical significance was determined by Students t- test. *p<0.05, **p<0.01.

The ability to adhere to, invade and replicate in eukaryotic cells, notably macrophages, is intimately associated with the pathogenesis of ssp. I serotype Typhimurium [24], and is important for virulence and systemic colonization by this organism [25], [26]. We postulated that the defect of Δ*tatC* mutants in systemic colonization after intraperitoneal infection might be due to a defect in internalization and/or survival within macrophages. To test this hypothesis we compared the Δ*tatC* mutant (HA473), a derivative of wild type ATCC14028 (HA420), and an isogenic Δ*invA* (HA458) mutant for adherence, internalization by, and replication in J774-A.1 macrophages. In these assays, the Δ*tatC* mutant was defective in cell association and uptake when compared to wild type ATCC14028 (Figure 2A). The ability to adhere to cells and be phagocytized was restored when *tatC* was supplied *in trans* (Figure 2A). To rule out the possibility that the defect in Δ*tatC* mutant association and invasion of macrophages was the result of a motility defect, we repeated our experiments using centrifugation to force the bacteria into close contact with J774-A.1 cells. Despite this treatment, the Δ*tatC* mutant was still defective for cell association, and was internalized more poorly than the isogenic wild type ATCC14028 (Figure 2A).

We examined the replication of wild type and Δ*tatC* mutant bacteria in J774A.1 macrophages at 24 hours post-infection. Wild type bacteria replicated approximately 100 fold after 24 hours under either condition. The Δ*tatC* bacteria replicated similar to wild type, when lower levels of cell association and internalization were taken into account. From this data we can conclude that the Δ*tatC* mutant is poorly internalized by macrophages, but when it enters the host cell it is still capable of surviving and replicating.

### Typhimurium Δ*tatC* mutant poorly colonizes the intestinal tract

**Figure 3:**
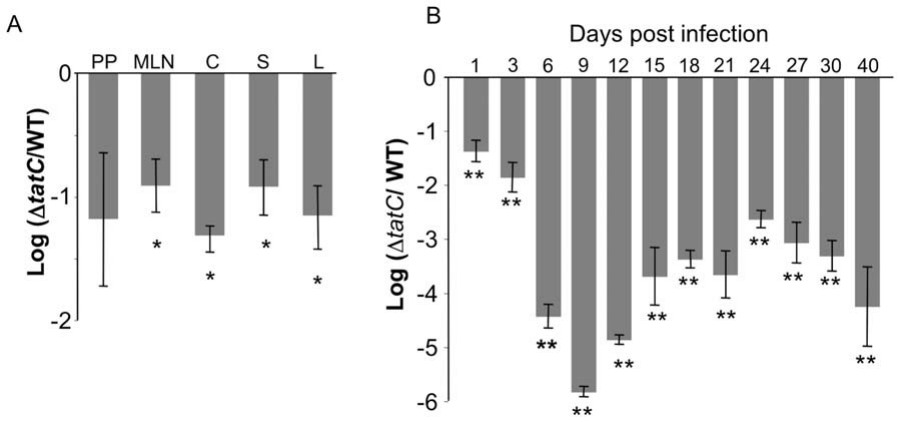
A Δ*tatC* mutant colonizes *Salmonella*-susceptible BALB/c mice and *Salmonella*-resistant CBA/J mice poorly after oral infection. (A) BALB/c mice were infected with an approximately equal ratio of wild type (HA431) and Δ*tatC* mutant (HA473) by oral inoculation using 2×10^7^ CFU. Mice were euthanized 5 days post infection and Peyer's patches (P), mesenteric lymph nodes (MLN), cecum (C), spleen (S), and liver (L) were collected for enumeration of the CFU of each infecting strain. Each bar represents the geometric mean for the infected mice of the ratio of mutant CFU to WT CFU collected from each organ, normalized to the ratio of the same in the inoculum, and converted logarithmically. Error bars denote standard error, and statistical significance was determined by Students t- test. *p<0.01. (B) Mice were infected with an equal ratio of wild type (HA431) and Δ*tatC* mutant (HA473) by oral gavage of 2×10^9^ bacteria. Fecal samples of six mice were analyzed over 40 days. Δ*tatC* mutants are heavily attenuated compared to wild type even at early time points. Each bar represents the geometric mean for the infected mice of the ratio of mutant CFU to WT CFU collected from each organ, normalized to the ratio of the same in the inoculum, and converted logarithmically, is shown. Error bars denote standard error, and statistical significance was determined by Students two-tailed t- test, **p<0.002.

We also assayed the ability of the Δ*tatC* mutant to colonize and persist in the intestinal tract of mice, both during short-term infections and for prolonged periods of time. In our short-term infections, we infected *Salmonella*-susceptible BALB/c mice orally with an equal mixture of wild type ATCC14028 and the Δ*tatC* mutant and evaluated colonization by both strains at 5 days post-infection. The Δ*tatC* mutant had a strong colonization defect in the cecum and systemic sites in this model (Figure 3A). The Δ*tatC* mutant did not appear to have a statistically significant colonization defect of Peyer's patches in our experiments. While this mutant may indeed have a defect in colonization of this tissue, we were not able to establish this due to high variability in colonization of this tissue in our experiments.

To evaluate colonization of the intestinal tract for longer periods of time, we infected and then followed fecal shedding of the Δ*tatC* mutant in *Salmonella*-resistant CBA/J mice. These mice possess a functional *NRAMP* allele, and do not develop systemic salmonellosis, although they are colonized by serotype Typhimurium in the gastrointestinal tract and this can be monitored by evaluating the level of fecal shedding [27]–[31]. We infected CBA/J mice orally with an equal mixture of the Δ*tatC* mutant and the isogenic wild type and monitored fecal shedding in these mice for 40 days post infection.

The Δ*tatC* mutant had 10-fold reduced fecal shedding from CBA/J mice at early time points post infection compared to wild type ATCC14028 (Figure 3B), consistent with our previous results showing reduced intestinal colonization by the Δ*tatC* mutant in BALB/c mice (Figure 3A). Furthermore, this defect became more severe with longer duration post infection (Figure 3B). By 30 days post infection the Δ*tatC* mutant was not detectable in the feces of most infected mice (3/5 mice). At 40 days, the Δ*tatC* mutant was undetectable in the feces of all 5 mice. The population remaining in the cecum at 40 days post infection overwhelmingly contained wild type *Salmonella* (data not shown), consistent with our fecal shedding data.

### Δ*tatC* mutant in Typhimurium ATCC14028 is highly sensitive to Bile Acids

Because the Δ*tatC* mutant was defective for intestinal colonization in murine models at very early time points after oral infection, we explored sensitivity to bile acids as a possible reasons for this phenotype. In *E. coli*, Δ*tatC* mutants are susceptible to detergent treatment due the inability to secrete *amiA* and *amiC* [21], [32]–[34]. *Salmonella enterica* serovar Enteriditis was recently shown to be susceptible to SDS and EDTA [35]. We hypothesized that Typhimurium Δ*tatC* mutants may also be more susceptible to detergent killing.

**Figure 4:**
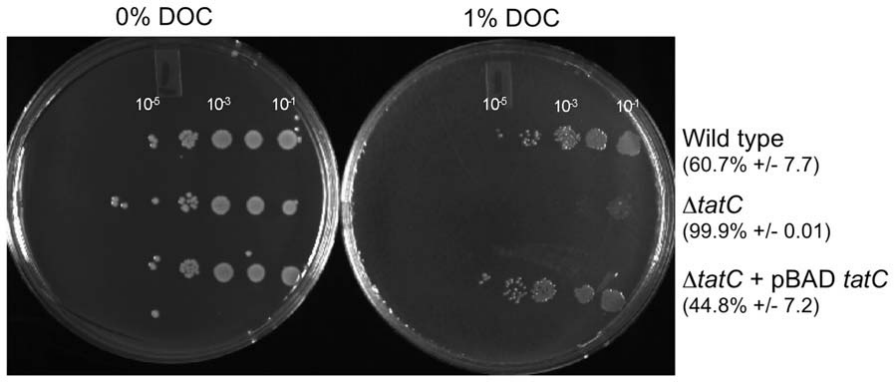
Δ*tatC* mutant is sensitive to bile acids. HA420 (ATCC14028 Nal^R^) and HA473 (ATCC14028 Nal^R^ Δ*tatC::*Kan^R^) were grown to stationary phase, subcultured and grown to exponential phase. Exponentially growing cultures (OD_600_ = 0.3–0.4) were serially diluted and spotted in 3 ml spots on LB agar or LB agar supplemented with 1% DOC. The Δ*tatC* mutant has reduced viability after treatment with 1% DOC. Samples shown are representative of three independent experiments, and percent killing as compared to growth on LB is noted.

We tested this hypothesis by evaluating the susceptibility of our Δ*tatC* mutant to the bile acid deoxycholate *in vitro* (DOC, 1%) [36]. Bacteria were grown in rich media overnight, sub-cultured in LB broth and grown to mid-log phase, and serial dilutions were plated on LB plates with or without 1% DOC. Both the wild type and the Δ*tatC* mutant grew equally well on LB plates without added DOC, yet the Δ*tatC* mutant had dramatically reduced survival on DOC as compared to the isogenic wild type (Figure 4, 99% killing of the Δ*tatC* mutant on 1% DOC). We show that the presence of a functional Tat system is responsible for this defect, as returning an intact copy of the *tatC* gene *in trans* restores the ability of this strain to survive 1% DOC at levels similar to the isogenic wild type. On this basis, we suggest that Δ*tatC* mutants may be sensitive to bile acid exposure during transit through the upper small intestine during infection. This poor survival of Δ*tatC* mutants in the upper small intestine may result in the defects in colonization of the lower intestinal tract, and reduced levels of fecal shedding that we show here.

### Altered motility of our Δ*tatC* mutant is a result of inability to express flagellins on the surface of *Salmonella*

**Figure 5:**
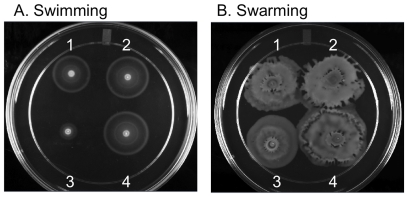
A Δ*tatC* mutant swarms, but swims poorly. Strains were plated on swimming (0.3% agar) and swarming (0.6% agar supplemented with 0.5% glucose) motility agar and incubated at 37°C for 5 and 7 hours, respectively. (1) wild type bearing empty vector (HA630), (2) wild type bearing the *tatC* complementing plasmid (HA634), (3) *tatC* mutant bearing empty vector (HA636), and (4) Δ*tatC* mutant complemented *in trans* (HA640). Assays were performed in triplicate on three separate occasions.

As part of a screen of a large number of mutants for motility defects, we also observed the Δ*tatC* mutant had a very unusual phenotype. This mutant is defective for swimming motility, but is able to swarm (Figure 5). Δ*tatC* mutant complemented *in trans* with *tatC* open reading frame had a restored ability to swim normally (Figure 5A). We incubated these mutants in various *in vitro* conditions and measured their ability to produce flagellins, FliC and FljB, and elaborate them on the bacterial surface.

**Figure 6:**
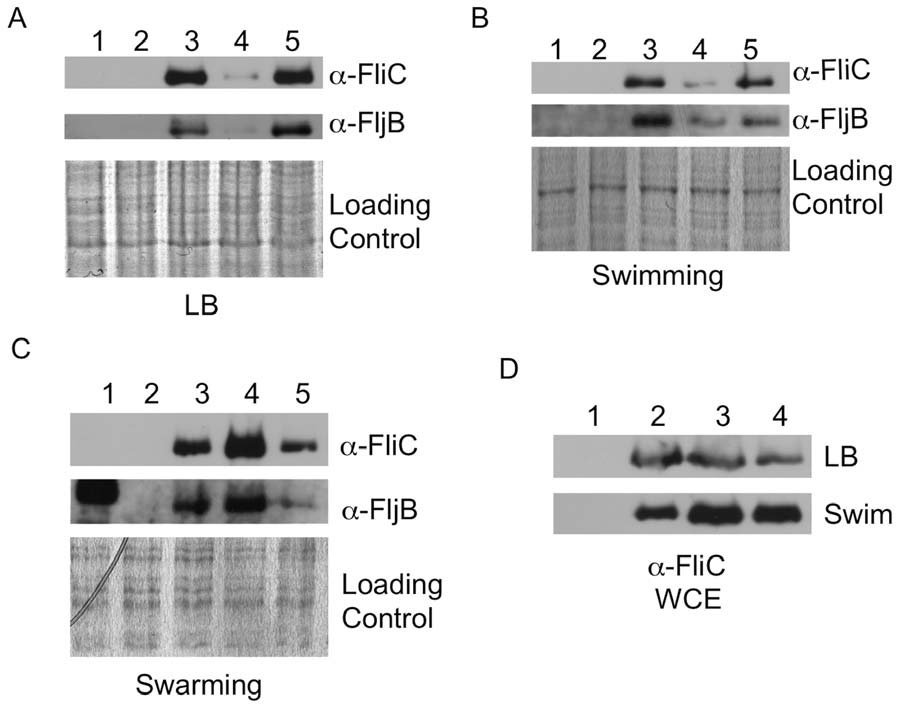
Flagellins in a Δ*tatC* mutant are decreased in LB and swimming conditions, but not in swarming conditions. Bacteria were harvested from LB broth, 0.3% agar plates used to assay swimming motility and 0.6% agar plates used to assay swarming motility as described in materials and methods. Flagellins sheared from the bacterial surface from strains grown in LB (Panel A), swimming conditions (Panel B), and swarming conditions (Panel C) were analyzed by Western blotting. Blots containing whole cell lysates are also shown (Panel D). Strains shown are: (Lane 1) HA478 (Δ*fliC*::Kan^R^), (Lane 2) HA690 (Δ*fljB*::Kan^R^), (Lane 3) Wild type (HA420, ATCC14028s), (Lane 4) Δ*tatC* mutant (HA473), and (Lane 5) Δ*tatC* mutant complemented *in trans* (HA640). Blots were probed with a-FliC and a-FljB polyclonal sera. The whole cell lysates for each sample were also analyzed by SDS-PAGE and stained with Coomassie Brilliant as a loading control. Blots shown are representative of three independent experiments.

Our wild type ATCC14028 derivative (HA420), elaborates the flagellins on the bacterial surface under all three conditions we tested: At stationary phase in LB broth, after incubation on swimming motility agar, and after incubation on swarming motility agar (Figure 6A, B, and C, lane 3). However, the Δ*tatC* mutant grown in LB broth or incubated on swimming plates had very little of either flagellin, FliC or FljB on their surfaces (Figure 6A and B, lane 4). The level of flagellins on the surface of the Δ*tatC* mutant was restored to the level of the isogenic wild type when the mutant was complemented *in trans* with an intact copy of *tatC* (Figure 6A and B, lane 5). Thus, the Δ*tatC* mutants had reduced motility on swimming motility agar as it likely produces fewer flagella in these conditions.

On swarming motility agar, the Δ*tatC* mutant is motile (Figure 5B, 3), and is able to elaborate flagellins on its surface when incubated on media for swarming motility (Figure 6C, lane 4). Thus, the mutant appears to produce flagella when swarming. Furthermore, the wild type and the Δ*tatC* mutant grown in LB and those incubated in conditions that allow swimming motility do produce flagellins that are present in whole cell lysate (Figure 6D). Our data show that the Δ*tatC* mutant can produce flagellins, and can elaborate flagellins on the bacterial surface under particular conditions. This data indicates that the lack of motility on swimming motility agar is not simply due to a blanket inability of the Δ*tatC* mutant to produce flagellins and assemble flagella on the bacterial surface.

### The predicted substrates of the TAT system in two closely related species are highly varied

Utilizing a genomic survey of prokaryotes we compared potential substrates of the TAT system in two closely related species, *Salmonella enterica* and *E. coli*. [37] Approximately 40% of the predicted substrates differ between the 2 organisms (Table 1), including 2 substrates, encoded on *Salmonella pathogenicity island 2* (SPI-2), but not part of the type III secretion system, *ttrA* and *ttrB*. Three substrates in *E. coli* were shown to have homology to duplicate genes in *Salmonella*, possibly due to large conserved domains in these enzymes (Table 1). A few protein sequences contain non-canonical signal sequences that were either predicted to be transported or shown experimentally. By comparing these two species, we can infer that even though they are closely related, the TAT system may have some overlapping and some distinct functions in both organisms.

**Table 1:** Comparison of potential TAT substrates in *E. coli* and *S. enterica* Serot. Typhimurium.

*Salmonella gene* #	*Salmonella gene* name	TAT signal (y/n)	*E. coli* gene #	*E. coli gene* name	TAT signal (y/n)	Function
STM0060	citE2^a^	y	b0616	citE	y	citrate lyase beta chain
**STM0070**	**caiD**	**n**	**b2919**	**scpB**	**y**	**methylmalonyl-CoA decarboxylase**
**STM0084**	**aslA**	**y**	**b3678**	**yidJ**	**n**	**putative sulfatase**
STM0107	thiP	y	b0067	thiP	y	thiamine transporter, ABC family
STM0168	cueO	y	b0123	cueO	y	multicopper oxidase
STM0193	fhuD	y	b0152	fhuD	y	hydroxamate dependent iron uptake
STM0611	ynfE^b^	y	b1587	ynfE	y	oxidoreductase
STM0622	citE a	y	b0616	citE	y	citrate lyase beta chain
**STM0834**	**ybiP**	**y**	**b0815**	**ybiP**	**n**	**putative integral membrane protein**
STM0964	dmsA	y	b0894	dmsA	y	dimethyl sulfoxide reductase, A
STM0996	ycbK	y	b0926	ycbK	y	putative outer membrane protein
**STM1383**	**ttrA**	**y**				**tetrathionate reductase (SPI-2)**
**STM1385**	**ttrB**	**y** **				**tetrathionate reductase (SPI-2)**
STM1498	ynfF	y	b1588	ynfF	y	putative dimethyl sulfoxide reductase
STM1499	ynfE^b^	y	b1587	ynfE	y	putative dimethyl sulfoxide reductase
STM1539	hyaA^c^	y	b0972	hyaA	y	hydrogenase 1, small subunit
STM1570	fdnG	y	b1474	fdnG	y	formate dehydrogenase
STM1622	ydcG/mdoD	y	b1424	ydcG/mdoD	y	glucans biosynthesis
**STM1710**	**pgpB**	**n** *	**b1278**	**pgpB**	**y**	**phosphatidylglycerophosphate phosphatase**
STM1786	hyaA^c^	y	b0972	hyaA	y	hydrogenase 1, small subunit
**STM2064**	**phsB**	**n**	**b1671**	**ydhX**	**y**	**putative oxidoreductase**
**STM2065**	**phsA**	**y**				**hydrogen sulfide production**
STM2099	wcaM	y	b2043	wcaM	y	putative colanic acid biosynthesis
STM2258	napG	y	b2205	napG	y	ferredoxin, electron transfer
STM2259	napA	y	b2206	napA	y	periplasmic nitrate reductase
**STM2446**		**n**	**b1019**	**efeB/ycdB**	**y**	**putative iron dependent peroxidase**
STM2450	amiA	y	b2435	amiA	y	N-acetylmuramoyl-L- alanine amidase I
STM2991	amiC	y	b2817	amiC	y	N-acetylmuramoyl-L- alanine amidase
**STM3058**	**pepP**	**y**	**b2908**	**pepP**	**n** *	**proline aminopeptidase II**
STM3149	hybA	y	b2996	hybA	y	putative hydrogenase
STM3150	hypO	y	b2997	hybO	y	putative hydrogenase
STM3172	sufI	y	b3017	sufI	y	suppressor of ftsI
STM3377	yedY	y	b1971	yedY	y	putative nitrate reductase
**STM3615**	**yhjK**	**n**	**b1163**	**ycgF**	**y**	**putative phosphodiesterase**
**STM3644**	**bisC**	**n**	**b1872**	**torZ**	**y**	**biotin sulfoxide reductase 2**
STM3822	torA	y	b0997	torA	y	TMAO reductase
STM4037	fdoG	y	b3894	fdoG	y	formate dehydrogenase
STM4190	pepE	y	b4021	pepE	y	alpha-aspartyl dipeptidase
STM4279	nrfC	y	b4072	nrfC	y	formate dependent nitrate reductase
STM4557	holD	y	b4372	holD	y	DNA polymerase III, psi subunit
**PSLT024**		**y**				**hypothetical protein**
**PSLT046**		**y**	**b0126**	**can**	**n**	**putative carbonic anhydrase**
**PSLT067**		**n**	**b0249**	**ykfF**	**y**	**hypothetical protein, CP4-6 prophage**
			b0286	yagT	y	**putative xanthine dehydrogenase**
			b0324	yahJ	y	**putative deaminase**
			b0705	ybfL	y	**pseudogene**

**Bold.** Differs between the two organisms.

a b c Gene duplication in Salmonella.

*twin arginine present, but not recognized as signal sequence.

**no twin-arginine, but shown to be a substrate (Hinsley et al. 2001)

Based on Dilks et al. (2003)

### Mutant Analysis of predicted TAT substrates

In order to determine whether one or more of the predicted substrates for *Salmonella* is responsible for the defect in motility, we generated deletion mutants for each of the substrates predicted by Dilks et al. [37]. Each of these mutants was assayed for ability to swim on swimming motility agar. Mutants in only one predicted substrate were observed to have a significant defect, Δ*STM4557* (*holD*). The *ΔholD* strain was unable to swim or swarm and Δ*holD* mutants exported flagellins to the bacterial surface poorly under all conditions tested (data not shown). Thus, if the phenotype of Δ*tatC* is due to a defect in transport, the transported protein or proteins responsible for the phenotype have yet to be identified.

### Deletion of RcsB partially restores motility to Δ*tatC* mutant

It has recently been shown that the master operon for flagellar synthesis, *flhDC*, is negatively regulated by *RcsB* [38]. In agreement with this finding, we have observed that deletion of *rcsB* leads to hypermotility (data not shown). The *Rcs* regulon is up-regulated in Δ*tatC* mutants in response to cell wall defects that can occur in these mutants [39].

**Figure 7:**
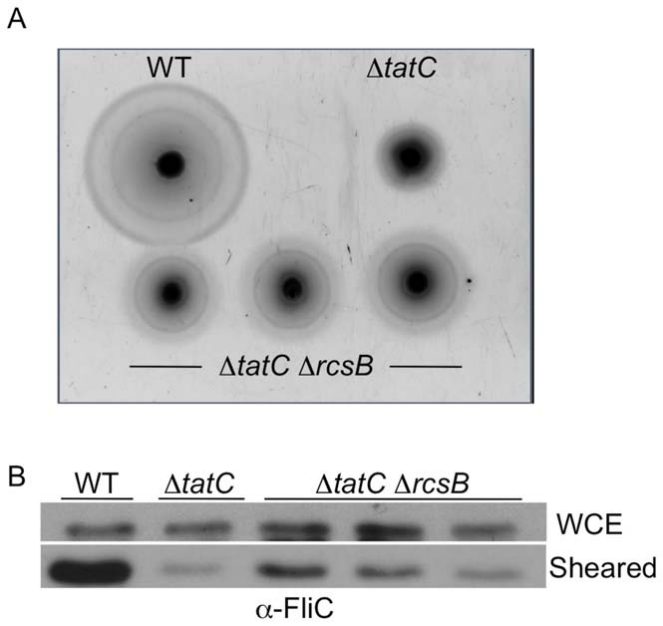
Deletion of *rcsB* partially restores swimming motility to a Δ*tatC* mutant. Wild type (HA420), Δ*tatC* mutant (HA473), and 3 independent colonies from a Δ*tatC*Δ*rcsB* double mutant (HA990) were examined for motility (Panel A) and flagellin elaboration on the bacterial surface (Panel B), as previously described. Deletion of *rcsB* in a Δ*tatC* background partially relieves the defect caused by Δ*tatC* alone.

In order to determine whether *rcs* is involved in the motility defect seen in Δ*tatC* mutants under swimming conditions, *rcsB* was deleted in a Δ*tatC* background. We compared the motility of the wild type parental isolate, the Δ*tatC* mutant, and a double Δ*tatC* Δ*rcsB* mutant on swimming motility agar (Figure 7). We hypothesized that if *rcsB* repression of *flhDC* is responsible for the motility defect, then deletion of *rcsB* should lead to a rescue of both swimming motility and surface flagellins. We observed that deletion of *rcsB* partially rescued the motility phenotype we observed in the Δ*tatC* mutant (Figure 7A). Furthermore, deletion of *rcsB* also partially rescued export of flagellins to the bacterial surface in the *ΔtatC ΔrcsB* double mutant (Figure 7B). Based on these findings and other work, we infer that relieving the *rcsB* repression of *flhDC*, allows *tatC* mutants to express flagellins on the bacterial surface and partially restores swimming motility.

## Discussion

Gram negative organisms secrete folded proteins across the inner membrane using the twin arginine transport (Tat) system. Disruption of the Tat system results in a range of defects in diverse processes including transport of virulence determinants, cell division, motility and chemotaxis [1]–[10]. In *E. coli* 0157:H7, Tat mutants have attenuated toxicity on Vero cells and motility defects [5]. In *Yersinia pseudotuberculosis*, a functional Tat system is necessary for virulence, motility and acid resistance [4]. In *Vibrio cholerae*, mutants in the Tat system colonize in the infant mouse model poorly and have reduced ability to form biofilms [10], but have normal motility. In *Pseudomonas aeruginosa*, an intact Tat system is necessary for transport of virulence determinants, such as phospholipases, and is also involved in iron uptake, stress defense, motility and biofilm formation [6], [7]. Some gram-negative organisms, such as *Mycobacterium tuberculosis*, rely on a functional Tat system for the secretion of beta-lactamases for antibiotic resistance [8], [9]. The number and complement of proteins predicted to be secreted by the Tat system is highly variable between organisms [37], and this may be one factor that influences the multiple and varied phenotypes of Tat mutants. Δ*tatB* mutants in *Salmonella enterica* serotype Enteriditis have recently been shown to colonize the cecum of 7-day old chick approximately 10 fold less efficiently in single infections [35], but no difference was seen in the ability of Tat mutants to colonize the systemic sites of chicks infected at either 4 or 7 days of age.

Tat mutants in *Salmonella enterica* serotype Typhimurium ATCC14028 also display multiple defects. We determined that a Δ*tatC* mutant in *Salmonella* is defective for growth in systemic sites after intraperitoneal infection, a route of infection that bypasses the intestinal tract. Because macrophages are an essential niche for serotype Typhimurium during systemic infection in mice [25], we tested the ability of the Δ*tatC* mutant to associate with, become internalized by, and replicate in macrophages. The ability of the Δ*tatC* mutant to associate with, be internalized by, and replicate in J774-A.1 was reduced as compared to the isogenic wild type.

Tat mutants in other pathogenic bacteria also associate poorly with or replicate to lower numbers in eukaryotic cells. In *Vibrio cholera*, mutants in the Tat system attach poorly to HT-29 cells [10]. *Legionella pneumophila* Tat mutants have defects in intracellular replication within differentiated U937 cells during later stages of growth [40]. Mutants in *tatB* and *tatC* in serotype Enteritidis are less invasive for polarized Caco-2 cells [35]. *E. coli* Δ*tatC* mutants have been have a cell septation phenotype and form long chains of bacteria [32], and a similar phenotype has been observed for Δ*tatC* mutants in *Salmonella* serotype Enteritidis [35]. Thus, the reduced internalization of *Salmonella* Tat mutants by cultured macrophages may actually be a difficulty in phagocytosis, as long chains of bacteria may not be easily internalized by macrophages.

We also show that a Δ*tatC* mutant is defective for intestinal colonization after oral infection of both *Salmonella*-susceptible BALB/c mice and *Salmonella*-resistant CBA/J mice. Similar defects in intestinal colonization have previously been shown for Tat mutants in other gram negative pathogens including *Vibrio cholera* [10] and *Yersinia pseudotuberculosis* [4]. Tat mutants in *E. coli* and *C. jejuni* have also been shown to have increased sensitivity to detergent and choleate [32]–[34], [41], [42]. We further show that a Δ*tatC* mutant in *Salmonella* is extremely sensitive to 1% Deoxycholic acid (DOC) *in vitro*, an assay commonly used to test for increased sensitivity to bile acids [36]. This sensitivity is likely a contributing factor for the reduced ability of *Salmonella* Δ*tatC* mutants to colonize the murine intestine.

Finally, similar to Tat mutants in other organisms [4]–[6], , this *Salmonella* Δ*tatC* mutant has a strong defect in swimming motility, as a result of failing to export flagellins to the bacterial surface under conditions that allow swimming motility. We show that a Δ*tatC* mutant is able to export flagellins to the surface and that they are motile under conditions that promote swarming motility. Thus, the defect in swimming motility displayed by a *Salmonella* Δ*tatC* mutant is not due to a general inability to export flagellins to the bacterial surface. Our *Salmonella* Δ*tatC* mutant seems to be unable to elaborate flagella on the surface under particular conditions. Similarly, Tat mutants in several other organisms, including *Agrobacterium* and *Pseudomonas*, were shown to be non-motile but to be able to produce flagella in some proportion of the swimming population [6], [44]. To our knowledge this is the first description of differential elaboration of surface flagellins in Tat mutants.

The mechanism responsible for the inability of Tat mutants to produce flagella in swimming conditions remains to be determined, but several hypotheses have been proposed. In Δ*tatABC* mutants of *E. coli* O157:H7 the inability to export FliC is hypothesized to be due to impaired insertion of FliP into the outer membrane [5]. The FliOPQR operon encodes class 2 flagellar proteins that make up part of the MS ring of the flagellar export system, and they are necessary for the export of flagellins [45]. FliP contains a signal sequence (M**RR**LLFLSLAGLWLFSPAAAA) necessary for its insertion into the cytoplasmic membrane and containing a twin arginine motif [46], [47]. However, the twin arginine motif in the FliP signal sequence is not recognized by either the TatP or TATFIND signal recognition software and FliP has never been shown experimentally to be secreted via the Tat system [37], [48].

Furthermore, elimination of FliP secretion when the Tat system is inactivated seems an implausible mechanism for the defects in swimming motility and flagellar export of *Salmonella* Tat mutants under swimming conditions, because we show that these mutants are still motile and able to secrete flagellins in swarming conditions. FliR, which acts in conjunction with FliP for the export of flagellin [47] is up regulated in Δ*tatC* mutants in *E. coli* under anaerobic conditions [39]. The effect on flagellins appears to be coordinated with environmental cues, and is likely to be more complex than was previously hypothesized.

The integrity of cell envelope of Δ*tatC* mutants is impaired in *E. coli* [34], and *P. aeruginosa* [6], and we show that *Salmonella ΔtatC* mutants are sensitive to the detergent DOC (Figure 5). In Δ*tatC* mutants in *E. coli* the Rcs regulon is up-regulated presumably in response to cell envelope defects. This regulon has also been shown to inhibit *flhDC* leading to impairment of motility [38]. It is possible that the defect in motility of our Δ*tatC* mutant is an indirect effect of increased Rcs activity. We show that deletion of *rcsB* in a Δ*tatC* background partially rescues both the swimming defect and the flagellar export defect. One possible interpretation of this data is that under swimming conditions a signal is necessary to remove *rcsB* from the promoter region of *flhDC* and this signal is not necessary under swarming conditions. When Δ*tatC* bacteria are shifted from swarming to swimming, they are able to reach a larger diameter in less time (data not shown), perhaps indicating that *rcsB* is not inhibiting *flhDC* under swarming conditions.

In summary, we show that a Δ*tatC* mutant in *Salmonella enterica* serotype Typhimurium ATCC14028 does not survive well in the intestinal tract or in systemic sites during experimental infections in mice. Our Δ*tatC* mutant is able to swarm, but is defective for swimming motility, an unusual phenotype. We show evidence for the inability to elaborate flagellins to the surface of mutants under swimming conditions. Motility and flagellar defects may be linked to the Rcs regulon as an indirect effect of deleting *tatC*, but additional work is required to determine the link between Tat and Rcs, and elaborate additional factors that suppress flagellar production in Tat mutants under swimming conditions. The ubiquitious nature of the Tat system among pathogenic bacteria make the twin arginine transport system a viable target for drug development.

## Materials and Methods

### Bacterial strains, plasmids and growth conditions

The strains used in this study are listed in Table 2 and were derived from *Salmonella enterica* serovar Typhimurium ATCC14028. We generated a spontaneous nalidixic acid resistant derivative of ATCC14028, HA420, that is virulent and persistent in murine models [49]. All deletion mutants were generated by the method of Datsenko and Wanner [50]. We confirmed the location of the mutation as a deletion in *tatC* by PCR using flanking primers. Deletion mutants that were studied further were moved into a clean genetic background by P22 transduction.

**Table 2:** Strains.

	Genotype	Citation
HA420	WT 14028s Nal^R^	[49]
HA431	HA 420 Δ*phoN*::Kan^R^	[49]
HA458	HA420 Δ*invA*::Kan^R^	[49]
HA473	HA420 Δ*tatC*::Kan^R^	This work
HA478	HA420 Δ*fliC*::Kan^R^	This work
HA690	HA420 Δ*fljB*::Kan^R^	This work
HA630	HA420 pBAD TOPO	This work
HA634	HA420 pBAD TOPO *tatC* Carb^R^	This work
HA636	HA473 pBAD TOPO	This work
HA640	HA473 pBAD TOPO *tatC* Carb^R^	This work
HA990	HA437 Δ*rcsB*::Cm^R^	This work

Strains were routinely cultured in Luria-Bertani (LB) broth and plates, supplemented with 50 mg/L Nalidixic acid, 100 mg/L Carbenicillin and 50 mg/L Kanamycin or 20 mg/L Chloramphenicol where appropriate. For the detection of *phoN* expression, 20 mg/L of 5-bromo-4-chloro-3-indolyl-b-D-phosphate (XP) was added to LB agar plates. Strains were grown in the presence of 0.02% arabinose for over-expression of TatC by arabinose induction. 0.5% glucose was used for catabolite repression.

For infections in murine models, all strains were grown at 37°C with aeration to stationary phase in Luria-Bertani (LB) broth containing the appropriate antibiotics. Strains used for invasion assays were grown statically for 16 hours at 37°C in LB broth containing 0.3 M NaCl, these conditions have been described previously to promote SPI-1 expression (Galan and Curtiss 1990; Bajaj, Lucas et al. 1996). For deoxycholate (DOC) sensitivity assays, strains were grown overnight in LB, and sub-cultured at a dilution of 1∶100 in LB and grown until log phase was reached (OD = 0.3–0.4), then used in DOC sensitivity assay as described below [36].

We complemented our Δ*tatC* mutant *in trans* by cloning an intact *tatC* open reading frame into pBAD TOPO (Invitrogen). A fragment containing the *tatC* open reading frame was amplified by PCR using primers: *tatC* Forward 5′ GGGACCGTAAACATGGCTGTA 3′ and *tatC* Reverse 5′ CGGTTGTGTAAAGTCTTCAGT 3′. The 780 base pair PCR product was ligated in frame with the pBAD promoter as well as a C-terminal fusion to a V5 epitope and polyhistidine region by altering the stop codon (see underlined base pairs). The orientation and frame of the cloned fragment were determined by dideoxy sequencing using the primers: pBAD Forward (5′ ATGCCATAGCATTTTTATCC 3′) and pBAD Reverse (5′ GATTTAATCTGTATCAGGCT 3′).

### Systemic infection of mice by intraperitoneal infection

All experiments involving animals described in this work were carried out in strict accordance with the recommendations in the Guide for the Care and Use of Laboratory Animals of the National Institutes of Health. The protocols used here were approved by the Institutional Animal Care and Use Committee at Texas A&M University (Permit Numbers: 2008-205 and 2009-069).

Our Δ*tatC* mutant was tested for colonization of 8–10 week old female BALB/c mice (Jackson Labs) in competitive infections with virulent *Salmonella enterica* serotype Typhimurium ATCC14028 derivative HA431 [23], using the following protocol. Strains used as inocula were grown to stationary phase at 37°C with aeration and mixed in a 1∶1 ratio of Δ*tatC* (HA473) mutant to HA431 (ATCC14028 Nal^R^ D*phoN*::Kan^R^), and then diluted to 1×10^7^ CFU/ml in PBS. Inocula were serially diluted and titered for bacterial CFU to determine the exact ratio of both strains in the competitive infection.

Groups of five to six mice were inoculated intraperitoneally with approximately 0.5–1×10^6^ bacteria in 100 ml of PBS. Two days post infection mice were humanely euthanized and livers, spleens, and ceca of infected mice were excised and homogenized in 5 ml ice cold PBS. Organ homogenates were serially diluted and plated to determine the ratio of Δ*tatC* mutant CFU versus wild type HA431 CFU from the collected tissues of infected animals. Data are expressed as the ratio of Δ*tatC* mutant CFU versus the wild type HA431 CFU, normalized to the input ratio, converted logarithmically, and displayed graphically. Statistical significance was determined using a Student's *t*-test and a P values as described in the figure legends.

Competitive infections were also used for complementation studies, essentially as described above. The Δ*tatC* mutant was transformed with either pBAD (empty vector) to generate the strain HA636, or pBAD containing a functional *tatC* gene (HA640). These strains, as well as HA431 (wild type), were grown as described above. HA431 was mixed in a 1∶1 ratio with either HA636 or HA640, and mixed inocula were used to inoculate mice and analyze the colonization of various organs as described above.

### Cell association, invasion and intracellular replication

The ability of our Δ*tatC* mutant to associate with, be internalized by and replicate within J774-A.1 macrophages was tested using the following method. J774-A.1 cells were propagated in DMEM (Cellgro) supplemented with10% Fetal Bovine Serum (PAA), and plated at a density of 3.5×10^5^ cells per well in tissue-culture treated 24 well dishes for all infections. Bacteria used for infecting J774-A.1 macrophages were grown to stationary phase without aeration in LB broth supplemented with 0.3 M NaCl (Galan and Curtiss 1990; Bajaj, Lucas et al. 1996). The titer of the inoculum in each experiment was determined by serial dilution and plating on appropriate bacteriologic media.

J774-A.1 cells were infected with *Salmonella* at a multiplicity of infection of 50∶1 (bacteria:macrophage), and bacteria were spun onto the cells at 750 rpm for 5 minutes where noted (Eppendorf 5804R). Infected cells were incubated for 1 hour at 37°C with 5% CO_2_ in a humidified tissue culture incubator. J774-A.1 monolayers were washed three times with 1 ml sterile PBS prior to lysis, to enumerate cell associated bacteria, or treated with 100 mg/ml gentamicin sulfate for 1 hour at 37°C with 5% CO_2_ in a humidified tissue culture incubator. For enumeration of intracellular bacteria that were internalized by J774-A.1 cells, gentamicin was removed and monolayers were washed three times with 1 ml sterile PBS. Infected monolayers were lysed in 1% Triton X-100, and intracellular CFU were enumerated by serial dilution and plating.

For assessment of intracellular growth, infected, gentamicin treated monolayers were washed with sterile PBS, and fresh DMEM supplemented with 10% FBS and 10 mg/ml gentamicin were incubated 24 hours post gentamicin treatment, washed with sterile PBS three times, lysed and intracellular CFU were enumerated. At each stage when infected cells were lysed, the number of viable J774-A.1 cells in duplicate infected monolayers was assessed by 0.4% Trypan Blue exclusion and counting viable cells. Each experiment evaluated samples in triplicate, and each experiment was performed on three separate occasions.

### Oral Infection in *Salmonella*-susceptible BALB/c mice and *Salmonella*-resistant CBA/J mice

For these experiments strains used as inocula were grown and mixed as described for systemic infections. Groups of four to six female BALB/c mice (8–10 weeks of age) were orally inoculated with approximately 2×10^7^ bacteria in 200 ml of LB. Infected mice were observed daily for signs of illness and were euthanized after the development of signs, at 5 days post-infection (inactivity and reluctance to move, ruffled fur, crouching together). Immediately after euthanasia, livers, spleens, Peyer's patches, mesenteric lymph nodes, and ceca of infected mice were excised and homogenized in 5 ml ice cold PBS. Organ homogenates were serially diluted and plated to determine the ratio of Δ*tatC* mutant CFU versus wild type HA431 from all of the collected tissues of infected animals.

Our Δ*tatC* mutant (HA473) was also tested for the ability to persist in the intestine of *Salmonella*-resistant CBA/J mice in competitive infections with HA431. 8–10 week old CBA/J mice were infected by gavage with an equal mixture of Δ*tatC* mutant and HA431, approximately 2×10^9^ in 100 ml LB in groups of 4–6 mice. Approximately 100 mg of feces were collected every three days, resuspended in 5 ml of sterile PBS, serially diluted and plated for enumeration of CFU of Δ*tatC* mutant vs. wild type HA431. After 40 days, mice were euthanized and livers, spleens, Peyer's patches, mesenteric lymph nodes, and ceca of infected mice were excised and homogenized in 5 ml ice cold PBS. Organ homogenates were serially diluted and plated to determine the ratio of Δ*tatC* mutant CFU versus wild type HA431 from all of the collected tissues of infected animals.

Data for infection experiments are expressed as the ratio of Δ*tatC* mutant CFU versus wild type HA431, were normalized to the input ratio, converted logarithmically, and displayed graphically. Statistical significance was determined using a Student's *t* test and a P value of <0.01.

### Deoxycholic acid sensitivity

Sensitivity to bile acids was estimated by assaying sensitivity to 1% deoxycholic acid [36]. Exponentially growing cultures (OD_600_ = 0.3–0.4) were serially diluted and spotted in 3 ml spots on LB agar or LB agar supplemented with 1% DOC. Plates were incubated overnight at 37°C.

### Motility Assays

Strains were tested for ability to swim on 0.3% agar Luria-Bertani (LB) plates or swarm on 0.6% agar LB plates supplemented with 0.5% glucose, as previously described [51]. Strains to be tested were spotted in equal amounts on the appropriate agar and incubated at 37°C for 5 or 7 hours, respectively and examined for motility.

### Flagellin precipitation and Western Analysis

Flagellin production was analyzed under a variety of conditions. Strains were initially grown in 5 ml LB broth to stationary phase. A portion of the culture was used for swimming and swarming motility assays, while the remaining cells from the culture were collected by centrifugation. Swarming bacteria were collected from swarming motility plates by swabbing the surface using a sterile cotton swab, resuspended in sterile PBS and collected by centrifugation after determining the bacterial concentration by OD_600_. Organisms were collected from plates used to assess swarming motility after 8 hours of incubation. Bacteria were collected from swimming motility plates by collecting agar plugs from inoculated swimming motility plates and the agar solids were removed by filtering using a sterile porous paper (Kimax) and gentle pressure. The number of bacteria in the filtrate was estimated by reading the OD_600_ and bacteria were collected by centrifugation.

The pelleted bacteria from all growth conditions were resuspended in 1 ml of sterile PBS and subjected to high- speed vortex for 5 minutes in order to shear the flagellins from the bacterial surface [52]. After centrifugation, the supernatant was removed and subjected to TCA precipitation (6% final concentration) on ice for 15 minutes [52], [53]. TCA precipitated proteins were collected by centrifugation at 4°C for 10 minutes at 13,000 rpm in an Eppendorf 5415R centrifuge and washed twice with 300 ml of acetone. Sheared precipitated proteins were resuspended in SDS-sample buffer. The bacterial pellet was also treated with 100 ml of SDS-sample buffer.

Sheared, TCA precipitated samples and whole cell lysates were boiled for 10 minutes in SDS-PAGE loading buffer, separated on SDS-PAGE (7.5% running gel, 4.5% stacking gel), and transferred to PVDF membrane using standard Western blotting protocols [54]. Membranes were blocked with 5% (wt/vol) non-fat dry milk in PBS containing 1% Tween (PBST) for 1 hour at 25°C and probed with rabbit α-FliC (Becton Dickson, Difco *Salmonella* H Antiserum i) at a dilution of 1∶10,000 or α-FljB (Becton Dickson, Difco *Salmonella* H Antiserum Single Factor 2) at a dilution of 1∶1,000 overnight at 4°C in blocking solution. Blots were washed three times in sterile PBST for 10 minutes. Alkaline phosphatase-conjugated secondary α-Rabbit IgG (Sigma) was used at 1∶10,000 in 5% (wt/vol) non-fat dry milk in PBST on blots for 1 hour at 25°C. Blots were washed three times in PBST followed by addition of the Immune-Star AP substrate (Bio-Rad) for 5 minutes.
